# Interventions to Decrease Overuse of Cardiac Monitoring (Telemetry) When Transitioning from the Intensive Care Unit to the Regular Nursing Floor

**DOI:** 10.7759/cureus.4311

**Published:** 2019-03-25

**Authors:** Johnny Chahine, Bicky Thapa, Falgun Gosai, Bahaa Abdelghaffar, Suleiman I Al Ashi, Anjli Maroo, Narendrakumar Alappan, K V Gopalakrishna

**Affiliations:** 1 Internal Medicine, Cleveland Clinic - Fairview Hospital, Cleveland, USA; 2 Cardiology, Cleveland Clinic - Fairview Hospital, Cleveland, USA

**Keywords:** cardiac monitoring, telemetry, quality improvement

## Abstract

Background

Cardiac monitoring (telemetry) is a common over-utilized hospital resource in the United States. Previous studies have shown that telemetry does not improve outcomes for low-risk patients. Inappropriate utilization occurs because of lack of awareness of guideline-based indications or non-adherence to known indications.

Objective

A quality improvement study was conducted to reduce telemetry overutilization during the transition of care from the intensive care unit (ICU) by 15% through increasing awareness of indications for telemetry.

Methods

The study cohort included patients originally admitted to the ICU for sepsis who had improved and were stable for transfer to a non-ICU setting. Subjects were identified and included during pre-intervention (six weeks) and intervention (six weeks) periods. Resident physicians and nurse practitioners were targeted using multiple modalities of education: didactic lectures during week one, poster demonstrations during week three, and video presentations during week five.

Results

A total of 246 study subjects during the pre-intervention and 94 study subjects in the intervention period were studied; 187 of the 246 subjects in the pre-intervention arm (76%) and 58 of the 94 subjects in the intervention arm (61.7%) were transferred with telemetry. Telemetry utilization dropped by 23.1% at the end of the intervention period.

Conclusion

Educating the caregivers about the indications for telemetry led to a decrease in over-utilization of telemetry on the transition of care from the ICU to the regular nursing floor. Repetitive and multi-modality educational interventions were effective tools and associated with increased adherence to established guidelines for telemetry usage.

## Introduction

Inpatient cardiac monitoring (telemetry) was introduced in the 1950s for the detection of arrhythmias. Since then, it has been widely utilized in the hospital setting. It has also helped monitor patients with acute coronary syndromes, titrate anti-arrhythmic medications, and determine the QTc interval. However, overutilization of telemetry causes alarm fatigue for nurses, increases the risk of delirium in elderly patients due to frequent awakenings and breaks in the sleep cycle, and raises the cost burden on patients, hospitals and the entire healthcare system [1]. Lack of awareness of appropriate indications for telemetry is the leading cause of overutilization. Cantillon et al. demonstrated that telemetry use with off-site monitoring improves outcome through early detection of abnormal heart rhythms [2]. However, telemetry is not beneficial in low-risk patients [3]. Improved physician awareness of the recent recommendations for telemetry use resulted in a decrease in telemetry overutilization [4]. We hypothesized that there could be a knowledge gap of those recommendations in our institution.

Solutions for the value enhancement (SolVE) cohort is a continuous quality improvement training program sponsored by graduate medical education of the Cleveland Clinic to pursue excellence in medical education. This 12-week program trains caregivers on how to successfully lead future quality improvement initiatives. Our SolVE team identified that telemetry is frequently ordered when the patients are transferred from the intensive care unit (ICU) (where all the patients are on cardiac monitoring) to the floor. Sepsis is a common admitting diagnosis to the ICU, and thus we decided to target this patient population. According to the recent recommendations, patients who have recovered from sepsis and are hemodynamically stable do not require cardiac telemetry when they are transferred from the ICU to the medical floors in the hospital [5]. We aim to decrease the number of sepsis patients transferred from the ICU to the regular nursing floor on telemetry by 15% within 6-8 weeks.

## Materials and methods

**Figure 1:**
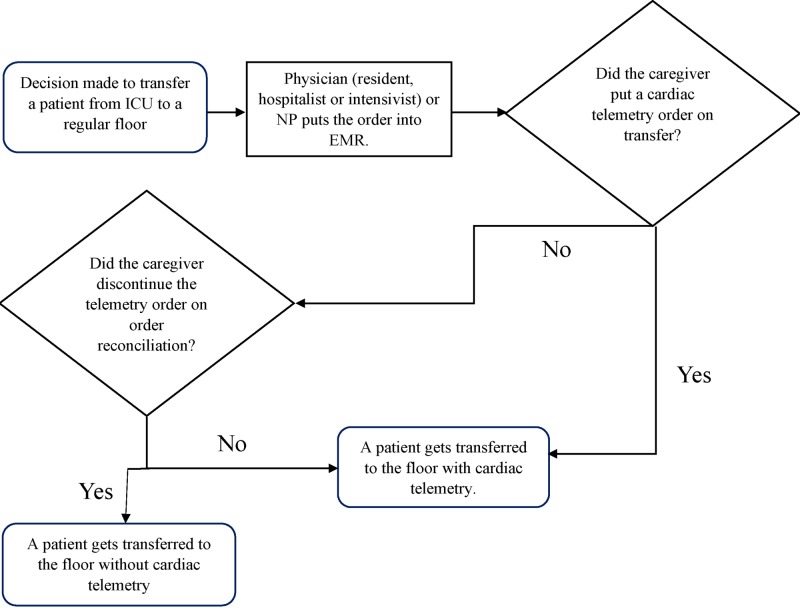
Process map depicting telemetry order placement while transitioning from the ICU to a regular nursing floor ICU: intensive care unit, NP: nurse practitioner, EMR: electronic medical record.

A team of five Internal Medicine residents participated in the SolVE project at the Cleveland Clinic Fairview Hospital. Data extraction using electronic medical records confirmed that telemetry is ordered in excess when transferring sepsis patients to the regular nursing floor. Study subjects included patients with sepsis who were stable and ready for transfer to a non-ICU setting. A process map was prepared to evaluate the telemetry order placement during the transition from the ICU (Figure 1).

A pre-implementation electronic survey was sent to the resident physicians, hospitalists, and intensivists to evaluate the familiarity of the caregivers with the indications of cardiac monitoring.

Tools used

Didactic Lectures

A PowerPoint presentation was prepared to emphasize the current status of telemetry utilization in the hospital, cardiac monitoring indications, and cost and the repercussion of its overuse on patients and nurses. Resident physicians, nurse practitioners, ICU nurses, and hospitalists were targeted during the first two weeks.

Posters

During the third week of the intervention, posters were distributed in the ICU and the residents’ and physicians’ lounges. The posters highlighted appropriate indications for telemetry, the positive impact of proper utilization, and the negative impact of overutilization of telemetry.

Video Presentation

A short video was prepared and featured two nurses narrating the patients’ discomfort caused by carrying the telemetry device and highlighting their increased risk for delirium because of frequent awakening at nights. They also spoke about the nursing alarm fatigue because of frequent artifacts and accidental disconnection of leads.

Methodology

Chart review and data abstraction for study patients were performed using the electronic medical record. In-hospital mortality was collected in intervention arm with and without telemetry. A measure of appropriateness of telemetry usage was calculated by dividing the number of study patients transferred from the ICU with a telemetry order by the total number of study patients transferred from the ICU to the regular nursing floor.

## Results

Pre-intervention results

A total of 246 study subjects who were transitioned from ICU to the regular nursing floor were identified in the six weeks before the intervention; 187 (76%) of these patients were transferred with telemetry. A pre-implementation online survey showed that 86% of caregivers were not familiar with the indications for telemetry on the regular nursing floor, which most likely contributed to the excessive orders for telemetry.

Intervention results

**Figure 2:**
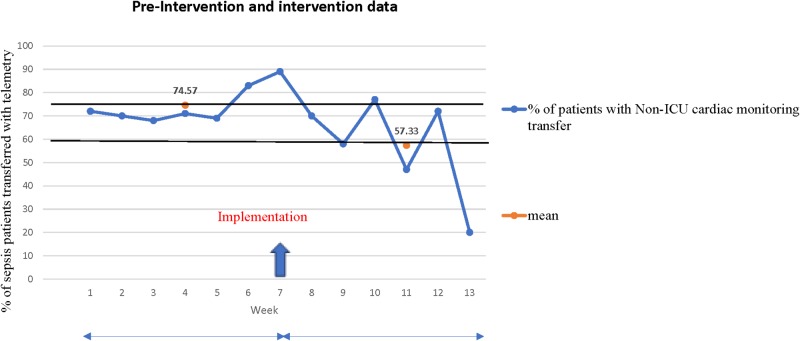
Graphical representation of pre-intervention and intervention data ICU: intensive care unit.

During the six-week intervention period, a total of 94 study subjects were transferred from ICU to the medical floor; 58 (61.7%) study subjects were transferred with telemetry during the intervention period. An incremental decrease in the telemetry utilization was noted in the consecutive weeks of the intervention period. During the first two weeks, there was a 2% reduction in telemetry use. In the following two weeks, there was a 21% reduction in telemetry use due to education reinforcement of appropriate indications for telemetry with poster illustrations. During the last two weeks, there was a 57% reduction in telemetry use after the video presentations. The mean percentage of study subjects transferred with telemetry was 74.57% before the intervention over a six-week period and 57.33% during the intervention over six weeks (Figure 2).

We were able to achieve a 23.1% reduction in telemetry utilization (target goal was 15%). There was no in-hospital mortality among study subjects in either group.

## Discussion

In 2004, the “American Heart Association Scientific Statement from the Councils on Cardiovascular Nursing, Clinical Cardiology, and Cardiovascular Disease in the Young” published guidelines regarding telemetry use, with class I indications recommending telemetry, class II indications favoring telemetry in certain conditions, and class III indications not endorsing cardiac monitoring [5]. Although hemodynamically unstable septic patients require cardiac monitoring (class I), septic patients who achieve hemodynamic stability do not need it when being transferred out of the ICU. Cardiac telemetry is inappropriately ordered throughout the hospitals in the United States [4,6]; telemetry was identified as one of the top five overutilized resources in hospitals by the Society of the Hospital Medicine [7]. Caregivers order cardiac telemetry with the belief that there will be better monitoring and prompt detection of lethal arrhythmias. However, Estrada et al. found that less than 1% of patients had life-threatening arrhythmias on cardiac telemetry [8]. Multiple factors contribute to the inappropriate use of cardiac monitoring such as non-adherence to guidelines, lack of awareness, and monitoring beyond the recommended time frame [3,9]. Overuse of telemetry causes patient discomfort and also negatively impacts nurses with artifacts and unnecessary alarms. Education is a very useful tool to increase awareness for the proper utilization of telemetry. We found that educating health care providers about the proper use of cardiac telemetry was an effective method to reduce healthcare costs in our hospital. Telemetry costs $230 per day in our hospital, and in a large-scale intervention, we anticipate a significant reduction in healthcare cost in addition to reduced patient discomfort and nursing alarm fatigue. It was necessary to utilize several forms of educational materials at different time points in our intervention to maximize adherence to established guidelines.

Limitations of our study are small sample size, the focus on subjects with sepsis, and short duration of the study to predict the outcome. Also, because this a single-center study with a small sample size, we could not assess for possible confounders. Other interventions such as incorporating the guidelines in the electronic medical record ordering system, removing the unnecessary telemetry order from order sets, and limiting the duration of the active telemetry order have proven to be beneficial in decreasing overutilization [4,10], and will provide additional sustainability of the results.

In the future, our team will need to establish longitudinal education campaigns to maintain adherence to established guidelines for telemetry use. We will need to broaden our educational efforts to include patients with additional diagnoses/conditions that do not require the use of non-ICU cardiac monitoring and extend our educational efforts to the emergency department. Finally, we would like to formally demonstrate that reducing inappropriate use of cardiac telemetry does not result in any compromise of patient safety or quality of care.

## Conclusions

Multiple education modalities that improve awareness of the appropriate indications for telemetry use is effective in decreasing inappropriate telemetry ordering during transfer from the ICU. This will result in reducing patient discomfort, nursing alarm fatigue, and hospital costs.

## References

[1] Clinical Alarms Task Force (2007). Impact of clinical alarms on patient safety: a report from the American College of Clinical Engineering Healthcare Technology Foundation. J Clin Eng.

[2] Cantillon DJ, Loy M, Burkle A (2016). Association between off-site central monitoring using standardized cardiac telemetry and clinical outcomes among non-critically ill patients. J Am Med Assoc.

[3] Henriques-Forsythe MN, Ivonye CC, Jamched U, Kamuguisha LKK, Olejeme KA, Onwuanyi AE (2009). Is telemetry overused? Is it as helpful as thought?. Cleve Clin J Med.

[4] Kanwar M, Fares R, Minnick S, Rosman HS, Saravolatz L (2008). Inpatient cardiac telemetry monitoring: are we overdoing it?. J Clin Outcomes Manag.

[5] Drew BJ, Califf RM, Funk M (2004). Practice standards for electrocardiographic monitoring in hospital settings. Circulation.

[6] Curry JP, Hanson CW, Russell MW, Hanna C, Devine G, Ochroch EA (2003). The use and effectiveness of electrocardiographic telemetry monitoring in a community hospital general care setting. Anesth Analg.

[7] Bulger J, Nickel W, Messler J, Goldstein J, O'Callaghan J, Auron M, Gulati M (2013). Choosing wisely in adult hospital medicine: five opportunities for improved healthcare value. J Hosp Med.

[8] Estrada CA, Rosman HS, Prasad NK, Battilana G, Alexander M, Held AC, Young MJ (1995). Role of telemetry monitoring in the non-intensive care unit. Am J Cardiol.

[9] Chen EH (2014). Appropriate use of telemetry monitoring in hospitalized patients. Curr Emerg Hosp Med Rep.

[10] Dressler R, Dryer MM, Coletti C, Mahoney D, Doorey AJ (2014). Altering overuse of cardiac telemetry in non-intensive care unit settings by hardwiring the use of American Heart Association guidelines. JAMA Intern Med.

